# Beyond endoscopic assessment in inflammatory bowel disease: real-time histology of disease activity by non-linear multimodal imaging

**DOI:** 10.1038/srep29239

**Published:** 2016-07-13

**Authors:** Olga Chernavskaia, Sandro Heuke, Michael Vieth, Oliver Friedrich, Sebastian Schürmann, Raja Atreya, Andreas Stallmach, Markus F. Neurath, Maximilian Waldner, Iver Petersen, Michael Schmitt, Thomas Bocklitz, Jürgen Popp

**Affiliations:** 1 Leibniz Institute of Photonic Technology, Jena, Germany; 2 Institute of Physical Chemistry and Abbe Center of Photonics, Friedrich-Schiller-University, Jena, Germany; 3 Institute of Pathology, Klinikum Bayreuth, Bayreuth, Germany; 4 Institute of Medical Biotechnology, Friedrich-Alexander University of Erlangen-Nuremberg, Erlangen, Germany; 5 Erlangen Graduate School in Advanced Optical Technologies (SAOT), Friedrich-Alexander University of Erlangen-Nuremberg; 6 Medical Department 1, Friedrich-Alexander University of Erlangen-Nuremberg, Erlangen, Germany; 7 Department of Internal Medicine IV (Gastroenterology, Hepatology, and Infectious Diseases), Jena University Hospital, Jena, Germany; 8 Institute of Pathology, Jena University Hospital, Jena, Germany

## Abstract

Assessing disease activity is a prerequisite for an adequate treatment of inflammatory bowel diseases (IBD) such as Crohn’s disease and ulcerative colitis. In addition to endoscopic mucosal healing, histologic remission poses a promising end-point of IBD therapy. However, evaluating histological remission harbors the risk for complications due to the acquisition of biopsies and results in a delay of diagnosis because of tissue processing procedures. In this regard, non-linear multimodal imaging techniques might serve as an unparalleled technique that allows the real-time evaluation of microscopic IBD activity in the endoscopy unit. In this study, tissue sections were investigated using the non-linear multimodal microscopy combination of coherent anti-Stokes Raman scattering (CARS), two-photon excited auto fluorescence (TPEF) and second-harmonic generation (SHG). After the measurement a gold-standard assessment of histological indexes was carried out based on a conventional H&E stain. Subsequently, various geometry and intensity related features were extracted from the multimodal images. An optimized feature set was utilized to predict histological index levels based on a linear classifier. Based on the automated prediction, the diagnosis time interval is decreased. Therefore, non-linear multimodal imaging may provide a real-time diagnosis of IBD activity suited to assist clinical decision making within the endoscopy unit.

Crohn’s disease (CD) and ulcerative colitis (UC) are the most common types of inflammatory bowel disease (IBD) with up to 12.7 and 24.3 of incidents per year for 100,000 individuals in Europe, respectively^1^. The severity of IBD and therefore, the adverse impact to the individual and the society can be reduced by appropriate medical treatment. In this regard, monitoring the response to therapy is a precondition for choosing the right therapeutics and the optimal dosage for individual patients. Traditional therapies of IBD result in an unspecific inhibition of inflammation with reduced clinical symptoms. Therefore, treatment end-points focused for many decades on the measurement of symptom severity, which shows only weak correlation with mucosal inflammation or disease related morbidity and mortality^2^. With the advent of biological therapies such as anti-tumor necrosis factor α (TNF) antibodies, which modulate specific pro-inflammatory pathways of IBD pathogenesis, the requirement for more reliable, well-defined end-points of therapeutic success became evident. Although these therapeutics show superior response even in patients with highly active disease, about 30% of patients initially do not response to anti-TNF treatment and further 10–50% of patients lose response after initial successful treatment in each subsequent year^3^. To allow a quick adaption of therapy in order to reduce the likelihood of acute flares or complications, it is necessary to identify non-responders as accurately and quickly as possible.

During recent years, endoscopic mucosal healing (MH) has been proposed as a superior end-point in comparison to clinical symptoms for the prediction of therapeutic success and the course of the disease. Endoscopic mucosal healing is defined as “complete absence of all inflammatory and ulcerative lesions” upon endoscopy and has been associated with a reduced progression of IBD and improved quality of life for affected patients^4^,^5^. However, due to inconsistent definitions of MH, problems with inter-observer reproducibility of endoscopic IBD scores, possible microscopic inflammation in endoscopically healthy mucosa and other factors, further improvements of end-points for IBD treatment are required. In this regard, histological remission has been proposed as an additional predictive marker for IBD progression and the response to therapy. Although histological remission has been used as an end-point for IBD therapy only in a very limited number of clinical trials, some studies could show a superior prediction of clinical relapse by histology in comparison to endoscopy or clinical symptoms in UC patients^6^,^7^. Due to a lack of adequate data, currently no conclusion can be drawn on the predictive value of histological remission in Crohn’s disease. As possible explanation for the lack of data on histological remission as an end-point for IBD trials might be the requirement of endoscopy to obtain biopsies and the associated risk for complications as well as the delay of diagnosis due to the processing and evaluation of biopsies.

In this regard, alternative techniques for the histopathological diagnosis of disease activity in IBD have to be developed to reduce the time spread until diagnosis. This might be achieved by real-time histological procedures *ex vivo* or even by *in vivo* endo-microscopy techniques. Over the past decade, it was increasingly recognized that non-linear multimodal imaging, the combination of coherent anti-Stokes Raman scattering (CARS), two-photon excited fluorescence (TPEF) and second harmonic generation (SHG), serve for accelerating diagnosis joining molecular contrast with subcellular resolution. In order to examine tissue sections, multimodal imaging was applied for the evaluation of human skin^8^, non-melanoma skin cancer^9^, larynx carcinoma^10^,^11^, brain tumors^12^, lung carcinoma^13^ as well as inflammatory bowel disease^14^. Recently, miniaturized multimodal setups were developed, which are suitable for application in a clinical environment such as gastroenterological endoscopy units^15^. For a future routine application of multimodal imaging within or next to an endoscopy unit, we have to show that the information in multimodal images are sufficient for IBD diagnostics.

For this purpose, we recorded 40 multimodal images of 20 tissue sections sized between 1 mm^2^ and 1 cm^2^ sampled from 20 patients with IBD. Five non-linear signals of the intestinal tissue were collected: two CARS channels corresponding to the two Raman resonances at 2850 cm^−1^ and 2930 cm^−1^, two TPEF channels detected at 426–490 nm and 503–548 nm and a further SHG channel. From all five modalities, various intensity and morphology related features were extracted and used to automatically predict different levels of chronicity, architecture and activity. Comparing automatic prediction results from multimodal images with the histopathological diagnosis, based on conventional H&E staining, showed a strong correlation of multimodal imaging with traditional pathology. Acquisition and evaluation of multimodal images of one patient optimally takes 5–10 minutes and therefore, allows a direct and timely diagnosis. These results show that multimodal imaging is a promising technique for the real-time evaluation of IBD activity that could assist clinical decision making.

## Results

**Figure 1:**
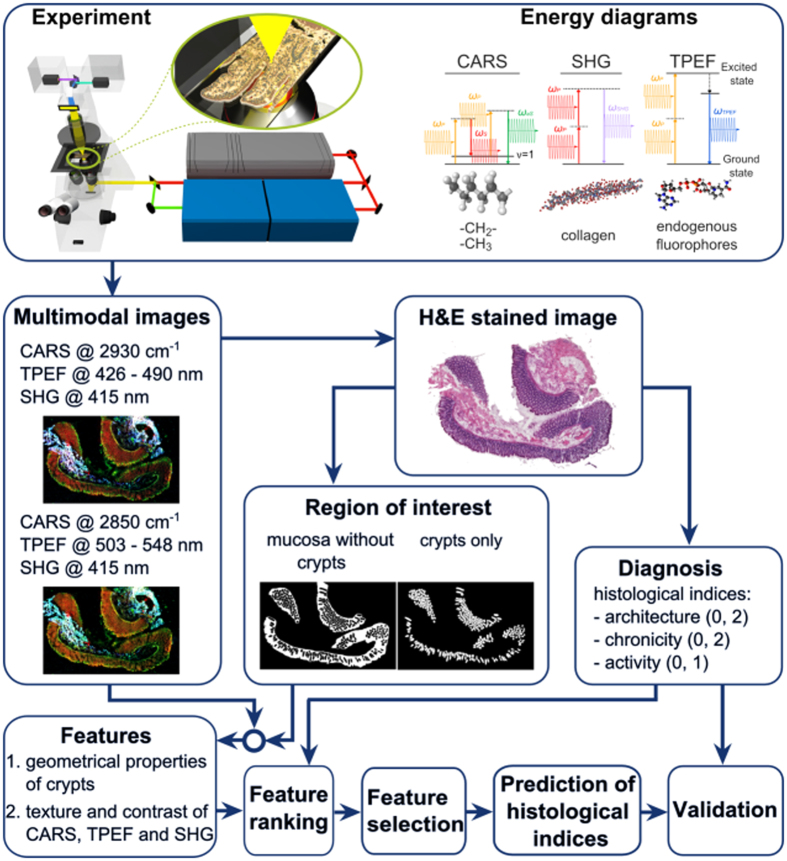
Workflow. 20 μm thick tissue sections of the intestinal tract are imaged by multimodal imaging. Each section is imaged twice combining (1) CARS @ 2930 cm^−1^, TPEF @ 503 nm – 548 nm, SHG and (2) CARS @ 2850 cm^−1^, TPEF @ 426 nm –490 nm, SHG. All sections are stained and regions of interest are selected and diagnosed by a trained pathologist. Using the multimodal images and the masks outlining regions of interest, morphology and intensity related features are extracted. These features are rated concerning their ability to discriminate indices of diagnostic criteria, i.e. architecture, chronicity and activity. The most significant features were chosen, analyzed and used for prediction of histological indices.

Within this study, multimodal images of 20 tissue sections taken from 20 patients were imaged and evaluated following the workflow presented in Fig. 1. The tissue sections with known IBD were imaged collecting five non-linear optical modalities: CARS at 2850 cm-1 (CARS2850), CARS at 2930 cm-1 (CARS2930), TPEF at 426–490 nm (TPEF458), TPEF at 503–548 nm (TPEF525), and SHG at 415 ± 0.4 nm. Afterwards, the sections were H&E stained and an experienced pathologist evaluated the sections based on the criteria given by Eyken *et al.*^16^ (see Table 1). This rating was used as histopathological gold-standard and within the study, we quantified the correlation of the multimodal images, e.g. the intensity and geometry related features, with these histopathological scores. As a correlation marker the FDR was utilized. Additionally, we demonstrated that a predictive model could be applied based on a LDA to predict the histological indexes from the multimodal images.

Visual evaluation of obtained images shows that multimodal images are able to display major indicators of an inflammation such as for example crypt distortions, lymphocytes, rupture of topmost epithelial layer, thickening of the basement membrane and scarring of the mucosa. As a result of a quantitative analysis, we found that the high accuracy of architecture index prediction can already be achieved by utilizing only geometrical properties of crypts, and that prediction itself can be carried out less sophisticated as compared to the chronicity and activity indices classification. The chronicity and activity indices can be only distinguished if intensity-related properties of CARS and TPEF are additionally taken into account, which is shown in the upcoming sections.

### Qualitative Characterization of the Intestinal Tract using Multimodal Imaging

**Figure 2:**
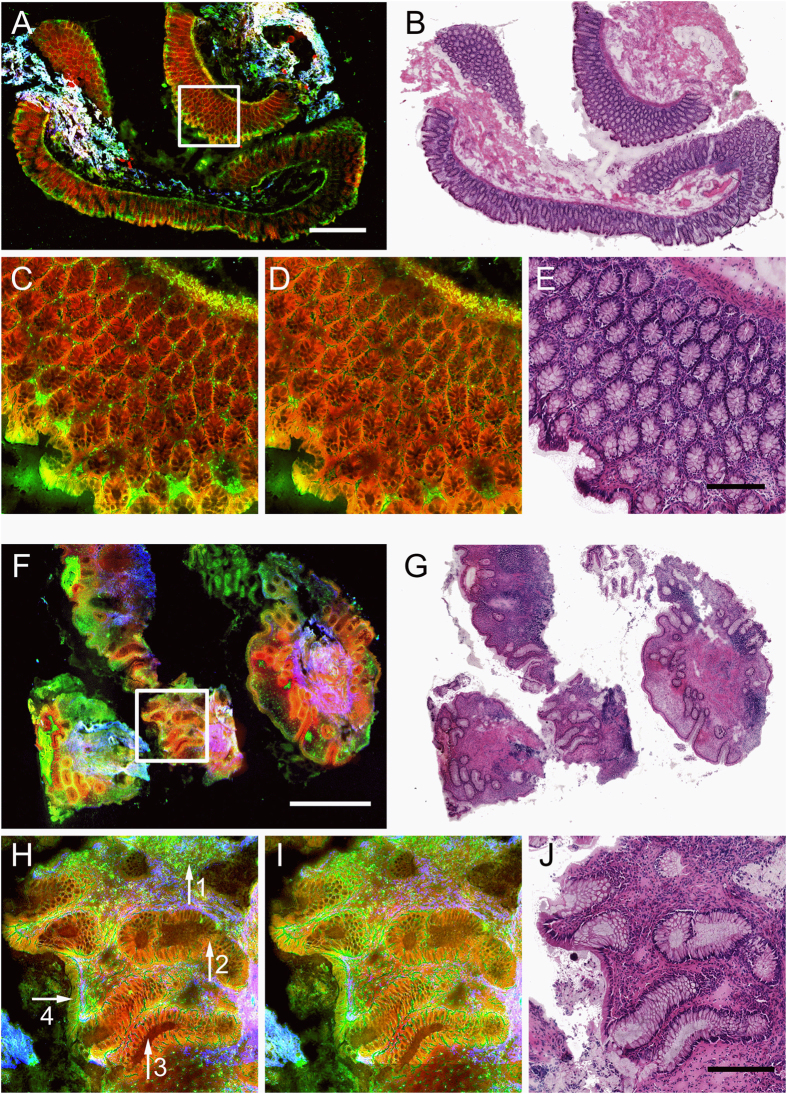
Multimodal images (**A,C,D,F,H,I**) and corresponding H&E stained tissue sections (**B,E,G,J**) of normal (**A–E**) and diseased (**F–J**) colon mucosa. Sub-figures (**A**, normal and **F**, ulcerative colitis) display large area multimodal images acquired with the combination of CARS @ 2930 cm^−1^, TPEF @ 503 nm – 548 nm and SHG that were diagnosed as architecture 0, chronicity 0 and activity 0 and architecture 2, chronicity 2 and activity 1, respectively. Sub-figures (**C**,**H**) show details of sub-figures (**A**,**F**) as outlined by white boxes. For comparison, sub-figures (**D**,**I**) show multimodal images corresponding to sub-figures (**C**,**H**), respectively, that were acquired with the combination of CARS @ 2850 cm^−1^, TPEF @ 426 nm –490 nm and SHG. Color-code – red: CARS, green: TPEF, blue: SHG. The white and the black scale bare correspond to 1 mm and 250 µm, respectively. Arrows: 1– lymphocytes, increase overall fluorescence, loss of crypt density; 2– crypt branching; 3-irregularities of crypts’ shape; 4- flattened epithelial layer facing the lumen.

Within this study 20 tissue sections were investigated using non-linear multimodal microscopy consisting of five non-linear optical modalities shown in Table 2 that yield information about distinct molecular structures. As an example of the multimodal image contrast, Fig. 2 presents multimodal and corresponding H&E stained images of a healthy, Fig. 2(A–E), and a strongly inflamed human colon, Fig. 2(F–J). Morphology information well-known from conventional staining protocols can be retrieved visually. In Fig. 2(A), the first major layers of the colon consisting of the mucosa and submucosa are readily identified. Zooming into the mucosa, the *muscularis mucosae* and the *lamina propria* are observed in Fig. 2(C,D). Within the latter, regularly shaped and uniformly distributed crypts can be identified with subcellular resolution. Here, mucin stored in secretory vesicles appears dark in CARS and TPEF, which allows for discrimination of goblet cells from enterocytes. Between the crypts, but also found inside, various cells of the lymphatic system are indicated by bright spots of TPEF as apparent in Fig. 2(C) due to high flavin concentrations. Interestingly, a visual comparison between the H&E stained and multimodal images revealed that not all lymphocytes but primarily eosinophilic granulocytes are highlighted in TPEF 525, but less by the TPEF 458 channel.

As the major indicator of an inflammation, the density of lymphocytes increases. The rise in lymphocyte density is indicated by an elevated density of bright cells in TPEF 525 and TPEF 458, as evident from Fig. 2(F,H,I). Further, the metabolic activity of cells within the inflamed area increases, which is connected to an increase in NAD(P)H and other fluorophores concentration. The latter causes a general elevation in the TPEF signal contribution from the inflamed intestinal tract. In addition to alterations of TPEF intensity levels, important morphology alterations connected to a recurrent fast rebuilding mucosa following destruction are readily identified in Fig. 2(H,I). The crypts feature various irregularities including an alteration of the crypt shape, variable internal and external diameters of the crypts, increased variability in the inter-cryptal distances, a loss of crypt density (mucosal atrophy), branching of crypts and separation from the *muscularis mucosae*. Furthermore, the topmost epithelial layer is flattened compared to its healthy counterpart in Fig. 2(C). Scarring of the mucosa as well as thickening of the basement membrane are outlined in Fig. 2(H,I) by areas with significant contributions of SHG signal which was not observed for non-inflamed colon tissue.

To support the diagnosis by multimodal imaging, these features are grouped under the terms of ‘architecture, activity and chronicity’ – see the green box for definitions. These terms are scaled by a trained pathologist with 0 or 2 (architecture and chronicity) or just 0 or 1 (activity) as a sizeable contribution to the final diagnosis that also takes further aspects of the patient’s history into account. Here, all features ranked 0 characterize a healthy non-inflamed colon such as presented in Fig. 2(A–E), while the maximum indices were assigned a by trained pathologist to the tissue section displayed Fig. 2(F–J).

### Quantitative Characterization of the Intestinal Tract using Multimodal Imaging

The visual evaluation of the multimodal images is subjective, time-consuming and requires a tedious and routine work of trained pathologist for a final diagnosis. Therefore, an automatic scoring of multimodal images for judgment of the intestinal architecture, activity and chronicity was developed to assist pathologists’ decision making based on further measures derived from multimodal images.

As the basis for any automated scoring algorithm, a quantitative evaluation of properties of the non-linear optical images is presented in the following to provide insights into the construction of the classification model and to justify the quality of prediction results. For prediction of histological indices, various geometrical features and intensity related properties (IRP) were obtained from multimodal images of mucosal areas with crypts only or without crypts (see region of interest in Fig. 1). Intensity related properties include texture characteristics of the spatial arrangment within single modalities and the contrast between different modalities. A summary of all 87 investigated features is given in sup. Tables S1 and S2 including their definition. Additionally, we added an example of a small area together with texture feature images to sup. Figure 1.

Each individual feature was ranked according to its ability to discriminate different levels of histological indices. The ranking was performed using Fisher’s discriminant ratios (FDR) due to its computational simplicity and its independence of the type of class distribution^17^ (See Supporting Information section for further details). The FDR is a statistical score and shows how well a value (texture features, intensity features) can be used to discriminate two groups. It can be used to rate the extracted features with respect to their class separation ability. In our study large FDRs outline features that are utilized best for class separation and discrimination of histological index levels. The results of the calculation of the FDRs are summarized in Table 3, displaying the ten most significant features for discrimination of various levels of chronicity, architecture and activity.

**Figure 3:**
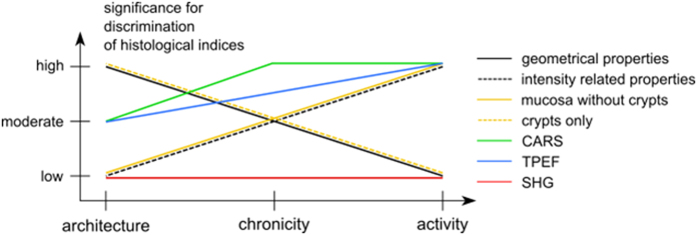
Trends of the significance of features for discrimination of histological indices. There is a clear trend for significance of geometrical properties of crypts, so that it is very important for architecture, less important for chronicity and unimportant for activity. The opposite trend is observed for intensity related properties (IRP). Further, the significance of IRP of crypts decreases from architecture to activity. IRP of mucosa without crypts is very important for activity and becomes irrelevant for architecture. Considering significance of non-linear optical modalities, TPEF and CARS are general important for histological indices discrimination, whereas SHG is insignificant.

Important results for the predictive modeling can be derived from the analysis of Table 3. These findings can be grouped into general trends that shall be summarized according to specific observations for histological indices (see Fig. 3). Comparing the maximum possible values of the FDR, it is observed that distinct levels of architecture (FDR_max_ = 4.41) shall be more simple to separate than for chronicity and activity with FDRs < 1.97. Consequently, a classification of the architecture levels can be treated computationally less elaborated than a prediction of the chronicity and activity.

**Figure 4:**
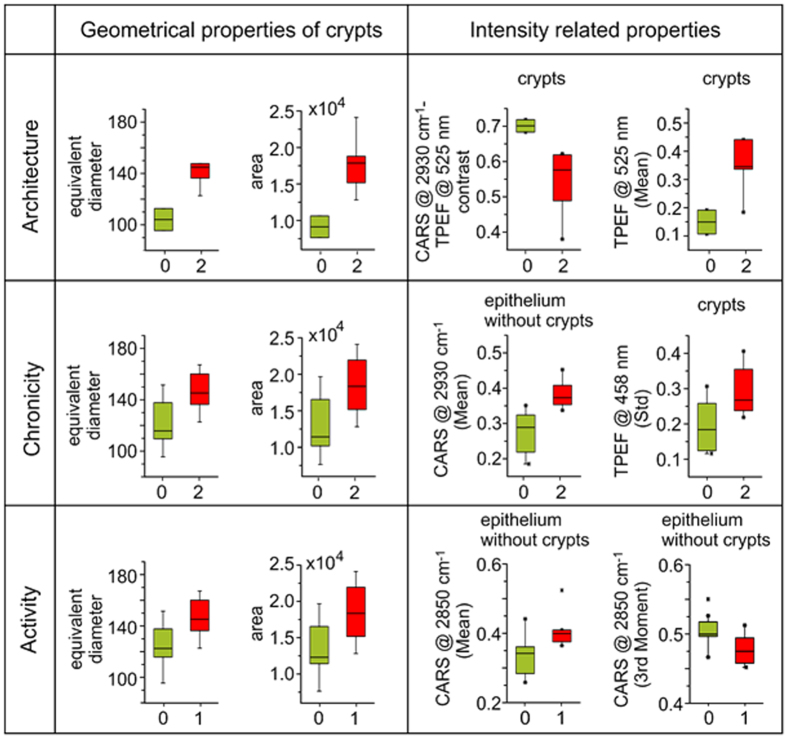
Boxplots of the two most significant geometrical properties and the two most significant intensity related properties for discrimination of architecture, chronicity and activity indices. Green color presents healthy samples (value of particular histological index is 0), red color indicates altered samples (value of histological index is 1 or 2). All histological indices were evaluated together, e.g. all samples with both UC and CD were utilized.

Further, the number of top-ranked geometrical properties of crypts is largest for the architecture index differentiation as expected from its definition as a measure of crypt distortion. Here, but also in general, the most significant geometrical features are the equivalent diameter, area, crypt density and perimeter of crypts. For visualization, the distributions for the equivalent diameter and the area of crypts are displayed for different levels of histological indices in Fig. 4. On the contrary, geometrical properties are less significant for the classification of chronicity and of lower significance for the classification of activity, as both criteria judge the presence of various types of lymphocytes and form measures of earlier or active inflammation, respectively. Note here, that the remaining significance of geometrical properties is no contradiction to the definition of chronicity and activity. Instead, the remaining importance reflects the correlation between chronicity and activity to the architecture that is constantly changed by the inflammation-induced rejuvenation of the colon.

Due to the reduced impact of geometrical properties, the IRPs are of highest significance for differentiation of chronicity and activity levels. Generally, it is observed that CARS- and TPEF-related measures are top-ranked, while no SHG-related feature was rated among the top-ten FDR values. Though the FDR values for SHG features of the architecture are close to zero, it shall be noted that its impact increased towards chronicity and activity. Among IRPs, the most significant features are identified as the mean and 3^rd^ moment of CARS@2850 cm^−1^, the mean of CARS@2930 cm^−1^, the CARS@2930 cm^−1^-to-TPEF@525 nm contrast as well as the mean and standard deviation of TPEF@525 nm and TPEF@458 nm as outlined in Fig. 4.

Furthermore, it is observed that the FDRs of areas comprising crypts only is highest for the architecture and decreases stepwise from the chronicity towards the activity in favor of the mucosal area excluding crypts. This observation is readily explained because an active inflammation starts outside crypts while a chronic IBD clearly affects crypts, and the architecture is the index of the crypts’ alteration.

Interestingly, it was found that TPEF collected around 525 nm is of higher impact for the evaluation of the activity and less for the architecture in comparison to TPEF collected at 458 nm while this trend reverses for chronicity. Similarly, it is observed that CARS at the Raman resonance 2930 cm^−1^ is more important for staging of the architecture and chronicity while for the activity, the significance of CARS at the Raman resonance 2850 cm^−1^ is more pronounced.

### Predictive Modelling of Histological Index Levels applying Multimodal Imaging

In the previous section, we used FDR values to quantify the class-separating ability of each individual feature independent from each other. In this section, we will build up a classification model to prove the possibility of an automated prediction of various levels of chronicity, architecture and activity providing further parameters that can assist the pathologist’s diagnosis.

The prediction of various levels of histological indices was performed utilizing a linear discriminant analysis (LDA). Three independent supervised classification models were developed, i.e. one each for architecture, chronicity and activity. The pathologist’s staging annotation was used as the reference for training of the LDA model. Out of the 87 features that were investigated in the previous section, a few tenths were selected to keep the model transparent and simple. Here, the feature selection was performed by finding the LDA classifier with best prediction accuracy as evaluated by one-leave-out cross validation. For each classifier tested, feature groups were evaluated, in contrast to the previous section where a scalar feature analysis was accomplished. As an advantage, this feature vector selection scheme enables for the investigation of the performance of several features at once in order to find the optimal combination for a particular classification task. It shall be noted that this selected feature combination may differ from a choice based on FDR values, as some features may measure similar or related properties of the colon, and their combination may not result in a significant improvement of classification results.

**Figure 5:**
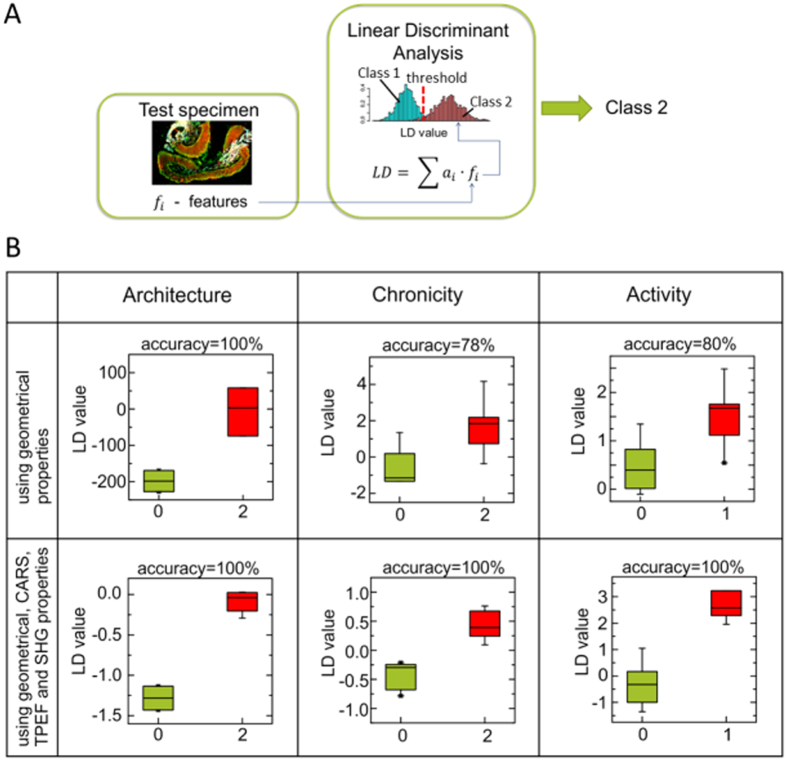
Automatic prediction of architecture, chronicity and activity levels utilizing LDA. Sub-figure (**A**) overview of a classification by a linear discriminant analysis (LDA). Geometrical and intensity related features of test samples are converted into LD values using a beforehand trained LDA model. The calculated LD values are compared with a threshold and a test sample is assigned to class 1 or class 2, respectively. Sub-figure (**B**) boxplots of LD values showing possibility of histological indices separation utilizing LDA. LD values are calculated using one-leave-out validation approach. Initially geometrical properties were implemented. Including of intensity related features has improved prediction accuracy. Green color presents healthy samples (value of particular histological index is 0), red color indicates altered samples (value of histological index is 1 or 2).

It was initially intended to use only geometrical properties for classification of all three histological indices. Indeed, an LDA based only on geometrical features achieves great classification results for the architecture index of colon tissue but yields moderate accuracies for the chronicity and activity, as evident from Fig. 5. To improve the classification of the latter, it was decided that IRP has to be included into the LDA model. Combining geometrical properties and IRP, nearly 20 features were selected for prediction of chronicity and activity indices while 10 features were chosen for the architecture prediction. The result of the corresponding LDA is displayed in Fig. 5. As evident from Fig. 5, combining geometrical properties and IRP returns the desired improvement of classification.

The results of the index level prediction demonstrate that non-linear multimodal imaging reveals the inflammation-related molecular contrast of IBD tissues. In this regard, non-linear multimodal imaging provides additional valuable information that allows an improvement of disease activity classification and can assist pathologist’s diagnosis. One limitation of this study is that the mucosal and crypts areas were labeled by a trained pathologist facilitating the image processing and prediction. However, it is a tedious and routine task for pathologist and delays the diagnosis. Since the implementation of IRP on the entire sample does not allow prediction of histological indices with high accuracy, the automated determination of the mucosal and crypts areas is very important and will need to be further explored and validated in future studies.

## Discussion

An accurate diagnosis for IBD is a prerequisite for an adequate therapy. However, evaluating endoscopic mucosal healing as a predictive marker is restricted by the fact that there are no generally accepted scoring systems, inter-observer reproducibility is limited, and there might be microscopic inflammation in endoscopically healthy mucosa. In this regard, histological mucosal healing has been proposed as a superior predictive marker for IBD. However, due to the required tissue preparation and histopathological examination, a considerable time is required until a histological diagnosis is provided. In this regard, various attempts have been performed to reduce the time until a histopathological diagnosis can be obtained *ex vivo* or even *in vivo* with endo-microscopic techniques.

For instance, Neumann *et al.* have used endo-cytoscopy for the differentiation of mucosal inflammatory cells in IBD. In that study, infiltrating neutrophilic, basophilic and eosinophilic granulocytes as well as lymphocytes could be identified in the mucosa and the amount of infiltrating cells showed a good correlation with histopathological scores^18^. However, endo-cytoscopy requires the topical administration of dyes, which is a time consuming task. Furthermore, experienced researchers are required that can interpret the data. Similarly, confocal laser endo-microscopy (CLE) has been used for the differential diagnosis of Crohn’s disease and ulcerative colitis, but not disease activity^19^. However, as CLE provides microscopic information based on the administration of fluorophores such as fluorescein, the obtained information is limited and does not provide comprehensive information about microscopic tissue morphology. In contrast, multiphoton microscopy-based detection of TPEF and SHG signals has been shown to provide a more detailed information about IBD-related microscopic changes of tissue morphology^14^.

Within the presented study, tissue sections from 20 patients with an established diagnosis of IBD were investigated using non-linear multimodal microscopy. As an advance to previous studies that were only based on the detection of TPFE and SGH signals, we added CARS in this study. Moreover, an extensive image analysis was applied to the recorded multimodal images extracting geometry- and intensity-related quantitative features. It was demonstrated that a subset of these features is highly correlating with levels of the histological indexes architecture, chronicity and activity. In particular, geometry-related features showed highest significance for the rating of the intestinal architecture while intensity-related features are more suited to categorize levels of chronicity and activity (see Table 3 and Fig. 5B). These findings allow for predictive modelling of the histological index levels based on the multimodal images. The most significant features were utilized for the generation of a statistical model, which predicts the histological index levels of chronicity, architecture and activity directly from the multimodal images. For the generation of this statistical model, an LDA model was applied, which features the advantage that its prediction is observer-independent. The prediction performance of the LDA was optimal compared with the histological gold standard for all histological indexes tested. By applying image analysis, the multimodal approach presented within this study resolves issues originating from different experience levels of the examiners and therefore, improves both IBD diagnosis and treatment control. This approach could not only be used following biopsy acquisition to directly obtain a diagnosis, but could also be implemented into clinical endoscopy, once miniaturized non-linear multimodal imaging devices are available. Due to the non-invasive character of the acquisition methods and the results of the presented study, the described combination of multimodal imaging and image analysis can be used as tool for a fast, examiner-independent and direct extraction of additional information from tissue sections, which may be applied in an endoscope.

## Materials and Methods

### Samples

Human tissue biopsies from patients with Crohn’s disease (n = 6), ulcerative colitis (n = 7) or infectious colitis (n = 7) were obtained from colonoscopy or surgical resections. Patients included 13 males and 7 females of age 20–65 years.

### Multimodal Imaging

A comprehensive description of the experimental setup is provided elsewhere^20^. A scheme of the experimental configuration is shown in Fig. 1. A Coherent Mira HP Titanium-Sapphire (Ti:Sa) laser (Coherent, USA) is pumped at 532 nm by a continuous wave Neodymium-Vanadate laser with an average power of 18 W. At a repetition rate of 76 MHz, the Ti:Sa-laser pulses at 830 nm with temporal width of 2–3 ps (FWHM). The Ti:Sa-laser’s averaged power output of 3.5 W is split into two parts by a beam splitter. Without frequency conversion, the first part is used directly as the Stokes beam. Allowing for adjustment of pump wavelength, the second part is coupled into an optical parametric oscillator (OPO, APE, Germany). To visualize the CH_2_ groups at 2850 cm^−1^ or CH_2_ and CH_3_ groups at 2930 cm^−1^ with CARS modalities, the OPO is tuned to 671 nm and 668 nm, respectively. For power stabilization, a noise eater (LCC3112H/M, Thorlabs, USA) is placed just after the beam exit of the OPO. The pump and Stokes beams are spatially and temporally overlapped and coupled into a laser scanning microscope (LSM 510 Meta, Zeiss, Germany). Both lasers are focused at the sample by a 20× (NA 0.8) achromatic objective (Zeiss, Germany). Every sample is imaged twice. First, CARS at 2850 cm^−1^, TPEF at 426–490 nm and SHG are collected simultaneously by three photomultiplier tubes (PMT, Hamamatsu Photonics, Japan). In a second experiment, the pump wavelength is shifted and CARS at 2930 cm^−1^, TPEF at 503–548 nm and SHG are jointly detected. To collect multimodal images of the whole sample, mosaic area scans with up to 10 × 15 tiles were acquired. Each tile covers an area of 450 μm × 450 μm and is recorded with 2,048 × 2,048 pixels resolution as well as 1.6 μs pixel dwell time. Every tile scan is averaged twice requiring a total acquisition time per tile that does not exceed 32 s for a single combination of three modalities, e.g. CARS at 2850 cm^−1^, TPEF at 426–490 nm and SHG. At the sample, the average laser power was measured to 50 mW and 25 mW for the Stokes and pump beam, respectively. A discussion about the laser power applied and the potential non-linear as well as linear tissue photo-damage is provided elsewhere^8^.

Since the excitation wavelengths for TPEF 458 and TPEF 525 are nearly identical and the spectral fluorescence emission spectrum is broad, both TPEF channels collect signal from the same endogenous fluorophores. The most prominent autofluorophores of the human intestine are elastin^21^, collagen^22^, NAD(P)H and flavins^23^. Due to the distinct spectral windows imaged by TPEF 458 and TPEF 525 however, these fluorophores appear with different relative signal contributions within each channel which is most pronounced for NAD(P)H and flavins. While NAD(P)H possesses a peak emission at 465 nm that is collected mostly by TPEF 458, fluorescence photons arising from flavins centered around 530 nm are collected more efficiently by TPEF 525. Thus, TPEF 458 and TPEF 525 complement each other for their ability to visualize areas of high NAD(P)H and flavins concentrations which is in fact the major reason for imaging every sample twice. Note, however, that a multimodal microscope is readily constructed that is able to collect both TPEF channels at once. CARS and TPEF are complemented by SHG revealing the location of non-centrosymmetric structures. For tissue sections of the intestinal tract, SHG, therefore, exclusively localizes the fibrous collagen network.

CARS 2850 maps the distribution of methylene groups-which are particularly abundant in lipids-by visualizing the symmetrical stretching (SymS) vibration of CH_2_. For CARS 2930, i.e. images at the Raman resonance at 2930 cm^−1^, the molecular interpretation is more sophisticated. Various aliphatic Raman bands overlap around 2930 cm^−1^, such as the Fermi resonances (FR) (~2905 cm^−1^, ~2918 cm^−1^, ~2954 cm^−1^) of CH_2_, the FR (~2934 cm^−1^) of CH_3_ and the SymS vibration (~2920 cm^−1^) of CH^24^. Nevertheless, the aliphatic contribution from non-methylene groups rises in CARS 2930 as compared to CARS 2850. Thus, CARS 2930 favors proteins in comparison to CARS 2850 as proteins possess a lower ratio of CH_2_ to (CH_3_ + CH) groups compared to lipids^15^.

### Image Analysis

Image processing and analysis were performed using MATLAB R2014b running on a commercial Fujitsu’s workstation CELSIUS R930 (Intel(R) Xeon(R) CPU E5-2643 v2 @ 3.50 GHz, 96 GB RAM).

Prior to the image analysis and prediction, the multimodal images were preprocessed as follows. The images were median filtered and reduced in size applying a down sampling of a factor of 4. The down-scaled images were corrected for mosaicking artifacts arising from the uneven illumination^25^, followed by a contrast adjustment procedure. To distinguish various histological indices, two types of features were extracted from the multimodal images. The latter can be grouped into geometrical properties of crypts and intensity-related properties of non-linear optical signals, such as texture characteristics within single modalities, as well as the contrast between different modalities^11^. Intensity-related properties were evaluated separately for all crypts and for the epithelial mucosa without crypts as assigned by a trained pathologist. All extracted features were ranked applying the Fisher discriminant ratio criteria (FDR) with respect to their discriminatory power^17^ (see Supporting Information section for further details). The most significant features were chosen, analyzed and used for prediction of histological indices by a linear discriminant analysis (LDA)^26^.

### Ethical Considerations

The collection of samples was approved by the ethical committee and the institutional review board of the University of Erlangen and all patients gave written informed consent. The applied methods were in accordance with the approved guidelines.

## Additional Information

**How to cite this article**: Chernavskaia, O. *et al.* Beyond endoscopic assessment in inflammatory bowel disease: real-time histology of disease activity by non-linear multimodal imaging. *Sci. Rep.* **6**, 29239; doi: 10.1038/srep29239 (2016).

## Supplementary Material

Supplementary Information

## Figures and Tables

**Table 1:** Definitions (as noted by Eyken *et al.*^16^).

Histopathological indexes	Definition			
**Architecture**	Degree of crypt distortions (shape, density and arrangement).			
**Chronicity**	Degree of infiltration of *lamina propria* by lymphocytes and plasma cells.			
**Activity**	Presence of neutrophils within the *lamina propria* or within epithelial structures such as the surface epithelium, the crypt epithelium (cryptitis) and lumen (crypt abscess), and unequivocal epithelial cell damage^16^.			

**Table 2:** Molecular structures of tissue highlighted by non-linear optical modalities.

Non-linear modality	Detection of molecular structure
CARS @ 2850 cm^−1^	CH_2_/lipids
CARS @ 2930 cm^−1^	CH_2_/lipids, CH_3_/proteins
TPEF @ 458 nm (426–490 nm)	NADH, elastin, collagen
TPEF @ 525 nm (503–548 nm)	Flavines, elastin, collagen
SHG @ 415 nm	collagen

**Table 3:** The ten most significant features are given for discrimination of architecture, chronicity and activity indices according to FDR criteria.

Architecture		Chronicity		Activity	
Feature	FDR	Feature	FDR	Feature	FDR
equivalent diameter	4.41	CARS@2930 cm^−1^ (mean) of epithelium without crypts	1.97	CARS@2850 cm^−1^ (mean) of epithelium without crypts	1.02
area	3.93	equivalent diameter	1.18	CARS@2850 cm^−1^ (3^rd^ moment) of epithelium without crypts	0.64
CARS@2930 cm^−1^ – TPEF@525 nm contrast of crypts	2.65	area	1.05	TPEF@525 nm (3^rd^ moment) of epithelium without crypts	0.63
TPEF@525 nm (mean) of crypts	2.49	TPEF@458 nm (std) of crypts	1.02	equivalent diameter	0.53
perimeter	2.34	TPEF@458 nm (smoothness) of crypts	0.93	TPEF@525 nm (mean) of epithelium without crypts	0.52
crypt density	2.24	perimeter	0.92	area	0.47
TPEF@458 nm (smoothness) of crypts	2.19	radius	0.86	TPEF@525 nm (mean) of crypts	0.46
CARS@2850 cm^−1^ (3rd moment) of epithelium without crypts	2.12	TPEF@525 nm (uniformity) of crypts	0.84	CARS@2850 cm^−1^ (3^rd^ moment) of crypts	0.42
TPEF@525 nm (std) of crypts	2.06	CARS@2850 cm^−1^ (uniformity) of epithelium without crypts	0.74	perimeter	0.41
radius	1.93	TPEF@525 nm (mean) of epithelium without crypts	0.66	radius	0.39

High FDRs outline features with great potential for classification purpose. Geometrical features are marked in bold.
