# Supplementary material for: Beyond endoscopic assessment in inflammatory bowel disease: real-time histology of disease activity by non-linear multimodal imaging
Source: Sci Rep. 2016 Jul 13;6:29239. doi: 10.1038/srep29239 (PMC4942779; doi:10.1038/srep29239)
Supplement: Supplementary Information [file srep29239-s1.pdf]

Beyond endoscopic assessment in inflammatory bowel disease: real-time histology of disease activity by non-linear multimodal imaging

Olga Chernavskaia[1,2,†], Sandro Heuke[1,2,†], Michael Vieth[3], Oliver Friedrich[4,5], Sebastian Schürmann[4,5], Raja Atreya[6], Andreas Stallmach[7], Markus F. Neurath[5,6], Maximilian Waldner[5,6], Iver Petersen[8], Michael Schmitt[2], Thomas Bocklitz[1,2,*], Jürgen Popp[1,2*]

Affiliations:

[1] Leibniz Institute of Photonic Technology, Jena, Germany.

[2] Institute of Physical Chemistry and Abbe Center of Photonics, Friedrich-Schiller-University, Jena, Germany.

[3] Institute of Pathology, University Medical Center Bayreuth, Bayreuth, Germany

[4] Institute of Medical Biotechnology, Friedrich-Alexander University of Erlangen-Nuremberg, Erlangen, Germany

[5] Erlangen Graduate School in Advanced Optical Technologies (SAOT), Friedrich-Alexander University of Erlangen-Nuremberg

[6] Department of Medicine I, University Medical Center Erlangen, Erlangen, Germany

[7] Department of Internal Medicine IV (Gastroenterology, Hepatology, and Infectious Diseases), Jena University Hospital, Jena, Germany

[8] Institute of Pathology, Jena University Hospital, Jena, Germany.

[†] Both authors contributed equally.

Supplementary information

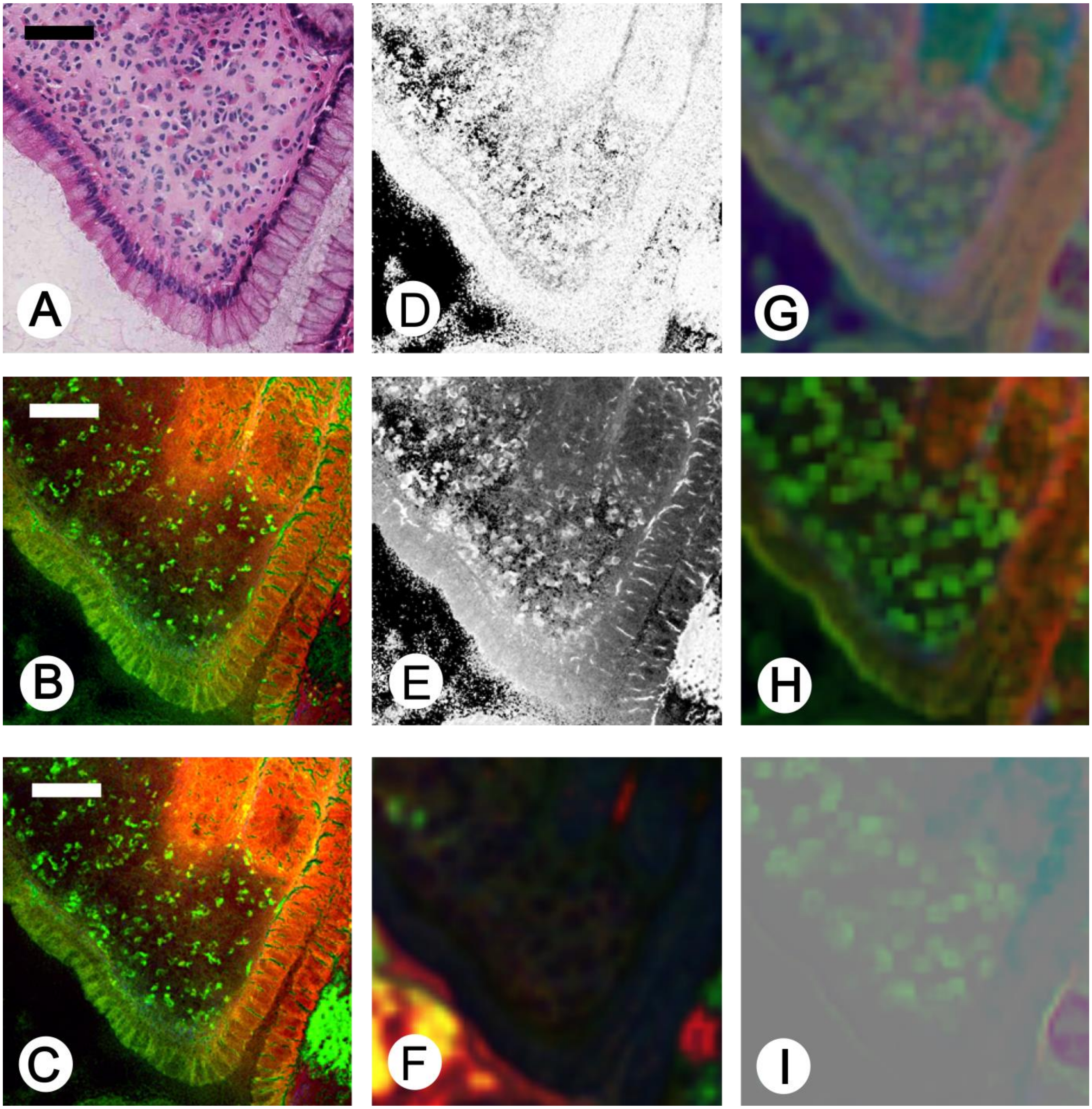

Supplementary figure 1. Invading immune cells including granulocytes. A – H&E, B – multimodal image (CARS@ 2850 cm^{-1}, TPEF@ 426 nm – 490 nm and SHG), C – multimodal image (CARS @ 2930 cm^{-1}, TPEF @ 503 nm – 548 nm and SHG), D – TPEF to SHG –contrast, E – CARS to TPEF contrast, F – uniformity texture image, G – entropy texture image, H –standard deviation texture image, I – 3rd moment texture image. The scale bare corresponds to 100 µm.

Supplementary information - Discussion: Quantitative Characterization: Fisher Discriminant Ratio (FDR)

Fisher´s discriminant ratio (FDR) is a class separating criteria that is commonly used due to its independence of the type of class distribution and computational simplicity[1]. Treating features individually, the FDR measures the classification capability with respect to a two-class problem in one-dimensional feature space. The FDR is defined as [1]

$$FDR = \frac{(\mu_1 - \mu_2)^2}{\sigma_1^2 + \sigma_2^2}, \quad (1)$$

where μ_1 and μ_2 are the mean values for first and second classes respectively and σ_1 and σ_2 the variance values of a particular feature. Treating features individually, the FDR measures the classification capability with respect to a two-class problem in one-dimensional feature space. High FDRs are obtained for a particular feature if the distribution of the two classes has small variances and display a large difference of mean values. This behavior is also obvious from the definition of the FDR in equation (1), which indicate that large differences between the mean values of each class and small variances within each class result in a high FDR. Thus, high FDRs imply a good class separation can be achieved utilizing a particular feature. The opposite scenario would be closely located classes with large class variance that would result in a low value of the FDR and hence in poor class separation.

Discussion: Quantitative Characterization: Geometrical properties of crypts.

Geometrical properties of crypts were extracted as follow. Firstly, a trained pathologist labeled crypts regions on HE image of particular sample. Secondly, labeled crypts regions were automatically recognized as separated segments. The properties of each segment were calculated and the mean value of each property over all segments was used as a feature for a particular sample.

Supplementary table S1. Geometrical properties of crypts

No	Feature	Description
1	area	the number of pixels in the crypt's region
2	aspect ratio	the ratio of the largest diameter to the smallest diameter
3	circularity	the ratio of crypt's area and square of perimeter
4	crypt density	number of crypts divided by epithelium area
5	eccentricity	the ratio of the distance between the foci of the equivalent ellipse and its major axis length
6	equivalent diameter	the diameter of a circle with the same area as the crypt's region
7	extent	the ratio of the pixel in the crypt's region to pixels on the total bounding box of the crypt
8	perimeter	the number of pixel of the boundary of the crypt's region

Supplementary table S2. Intensity related properties

No	Feature	Description
Contrast		
9-16	CARS – TPEF contrast	(CARS-TPEF)/(CARS+TPEF)
17-20	CARS – SHG contrast	(CARS-SHG)/(CARS+SHG)
21-23	TPEF – SHG contrast	(TPEF-SHG)/(TPEF+SHG)
24-25	CARS2850 – CARS2930 contrast	(CARS2850-CARS2930)/(CARS2850+CARS2930)
26-27	TPEF458 – TPEF525 contrast	(TPEF458-TPEF525)/(TPEF458+TPEF525)
Texture		
28-37	Mean	Average intensity
38-47	Standard deviation	Average contrast
48-57	Smoothness	Relative smoothness .
58-67	3th moment	Skewness of intensity histogram.
68-77	Uniformity	U is maximal when all intensity values are equal
78-87	Entropy	Variability of intensity

1. Theodoridis, S., Pikrakis, A., Koutroumbas, K. & Cavouras, D. *Introduction to Pattern Recognition: A Matlab Approach*. (Academic Press, 2010).
